# No clinically relevant QTc prolongation with ceftibuten or ledaborbactam etzadroxil: concentration-QTc analyses from two phase I studies

**DOI:** 10.1093/jac/dkag365

**Published:** 2026-10-23

**Authors:** Borje Darpo, Hongqi Xue, Kamal A Hamed, Karine Litherland, Marc Engelhardt, Kathryn Lowe, Paul C McGovern

**Affiliations:** Clario, part of Thermo Fisher Scientific, Philadelphia, PA, USA; Clario, part of Thermo Fisher Scientific, Philadelphia, PA, USA; Basilea Pharmaceutica International Ltd, Allschwil, Switzerland; Basilea Pharmaceutica International Ltd, Allschwil, Switzerland; Basilea Pharmaceutica International Ltd, Allschwil, Switzerland; Venatorx Pharmaceuticals, Inc., Malvern, PA, USA; McGovern ID Consulting LLC, Berwyn, PA, USA

## Abstract

**Objective:**

Ceftibuten and ledaborbactam etzadroxil are being developed as an oral fixed-dose combination for the treatment of complicated urinary tract infections caused by multidrug-resistant Enterobacterales. This study evaluated their effects on cardiac repolarization using concentration-QTc analyses.

**Methods:**

ECG and pharmacokinetic data from two randomized, single-centre, double-blind, placebo-controlled, phase I, dose-escalation studies in healthy adults (ClinicalTrials.gov: NCT04314206 and NCT04243863) were analysed using concentration-QTc modelling. Thirty-six participants were randomized to the ceftibuten study (27 active, nine placebo) and 48 to the ledaborbactam etzadroxil study (36 active, 12 placebo). Linear mixed-effects models characterized the relationship between plasma concentrations and placebo-corrected change from baseline in Fridericia-corrected QT interval (ΔΔQTcF). Model-based predictions estimated QTc effects across observed and clinically relevant plasma concentrations.

**Results:**

Neither agent produced clinically relevant changes in ECG parameters. Concentration-QTc analyses demonstrated no statistically or clinically meaningful relationship between plasma concentrations and ΔΔQTcF. An effect on ΔΔQTcF exceeding the regulatory threshold of 10 ms could be excluded at plasma concentrations up to 44 µg/mL for *cis*-ceftibuten (the predominant and more potent isomer), 2.2 µg/mL for *trans*-ceftibuten, 30.3 µg/mL for ledaborbactam (active drug), and 2.6 µg/mL for ledaborbactam etzadroxil (prodrug). A small, transient, not clinically relevant increase in heart rate was observed with ceftibuten, whereas ledaborbactam etzadroxil had no clinically meaningful effect on heart rate.

**Conclusions:**

Ceftibuten and ledaborbactam etzadroxil demonstrated no clinically relevant effects on cardiac repolarization or ECG parameters. Concentration-QTc analyses excluded clinically meaningful QTc effects at and above anticipated therapeutic plasma concentrations, supporting the cardiac safety profile of this oral fixed-dose combination.

## Introduction

The oral fixed-dose combination of ceftibuten and ledaborbactam etzadroxil (ceftibuten-ledaborbactam etzadroxil) is currently in development for the treatment of complicated urinary tract infections caused by multidrug-resistant Enterobacterales. Ceftibuten is a broad-spectrum, third-generation cephalosporin antibiotic approved in the United States and several European countries, with high oral bioavailability and activity against Enterobacterales.^1^ Ceftibuten exists as two isomers, *cis*- and *trans*-ceftibuten; *trans*-ceftibuten has substantially lower antimicrobial activity than *cis*-ceftibuten and accounts for approximately 10% of total ceftibuten exposure.^2^ Ledaborbactam etzadroxil (formerly VNRX-7145) is an oral prodrug of ledaborbactam (formerly VNRX-5236), a broad-spectrum β-lactamase inhibitor.^3^ Following single oral doses of 100 to 1000 mg, ledaborbactam etzadroxil was rapidly converted to ledaborbactam: prodrug exposure was ≤2% of ledaborbactam exposure, and the geometric mean terminal half-lives were 2.8 to 4.7 hours for ledaborbactam etzadroxil and 5.5 to 11.3 hours for ledaborbactam.^4^

Although neither ledaborbactam etzadroxil nor its active metabolite, ledaborbactam, has intrinsic antimicrobial activity, ledaborbactam restores the activity of ceftibuten against β-lactamase–producing Enterobacterales. *In vitro*, the combination of ceftibuten and ledaborbactam demonstrated potent activity against Enterobacterales, including multidrug-resistant isolates, with susceptibility exceeding 90% across multiple studies.^3,5,6^ The combination inhibits Enterobacterales producing class A extended-spectrum β-lactamases (ESBLs) such as cefotaximase-Munich (CTX-M) and sulfhydryl reagent variable (SHV), class C AmpC enzymes, and serine carbapenemases including class A *Klebsiella pneumoniae* carbapenemase (KPC) and class D oxacillinase-48 (OXA-48)-like enzymes.^3,5,6^ Ledaborbactam does not inhibit class B metallo-β-lactamases.

Ceftibuten has been reviewed by CredibleMeds; however, currently available evidence does not place it within any torsades risk category, as β-lactams are typically not associated with QT prolongation.^7^ Although β-lactam antibiotics are not generally associated with clinically significant QT prolongation, regulatory guidance recommends assessment of QT liability for new systemically administered drugs during clinical development. The International Council for Harmonisation (ICH) E14 guideline and its accompanying Questions and Answers, most recently revised in 2026,^8^ support concentration-QTc modelling in early clinical studies as the primary approach for evaluating QT/QTc interval prolongation and proarrhythmic potential for non-antiarrhythmic drugs.^9^ Here, we report concentration-QTc analyses evaluating the effects of ceftibuten and ledaborbactam etzadroxil on the heart rate–corrected QT interval (QTc) using pharmacokinetic (PK) and electrocardiographic data from these two phase I studies.

## Methods

### Ethics

This analysis used data from two previously conducted Phase I clinical studies in healthy adult volunteers. Both studies were approved by an institutional review board (VNRX-5024-101: Beoordeling Ethiek Biomedisch Onderzoek [Evaluation of Ethics in Biomedical Research], Assen, The Netherlands; VNRX-7145-101: IntegReview IRB, Austin, Texas, USA) and conducted in accordance with the ethical principles of the Declaration of Helsinki, the International Council for Harmonisation Good Clinical Practice guidelines, and applicable regulatory requirements. Written informed consent was obtained from all participants prior to screening.

### Participants

Cardiodynamic evaluation was performed in participants enrolled in the single-ascending-dose cohorts of two randomized, single-centre, double-blind, placebo-controlled, phase I, dose-escalation studies of ceftibuten (NCT04314206; VNRX-5024-101) and ledaborbactam etzadroxil (NCT04243863; VNRX-7145-101), the full designs and primary safety and PK results of which have been reported previously.^1,4^ Continuous Holter ECG recordings were obtained before dosing on day 1 and continued through 24 hours after dosing. In the ceftibuten study, 36 participants were randomized in a 3:1 ratio to receive a single oral dose of ceftibuten (400, 800, or 1200 mg) or placebo (ceftibuten, *n* = 9; placebo, *n* = 3 per dose level). In the ledaborbactam etzadroxil study, 48 participants were randomized in a 3:1 ratio to receive a single oral dose of ledaborbactam etzadroxil (100, 200, 300, 500, 800, or 1000 mg) or placebo (ledaborbactam etzadroxil, *n* = 6; placebo, *n* = 2 per dose level). One participant who received ledaborbactam etzadroxil was excluded from the concentration-QTc analysis as there was only one ECG observation at 24 hours post-dose and no pre-dose observations. Consequently, the analysis included 47 evaluable participants (35 who received ledaborbactam etzadroxil and 12 who received placebo).

### Endpoints and analyses

The primary objective of the cardiodynamic evaluation was to assess the effect of ceftibuten or ledaborbactam etzadroxil on the Fridericia-corrected QT interval (QTcF). Secondary objectives included evaluation of the effects of ceftibuten or ledaborbactam etzadroxil on other ECG parameters, including heart rate (HR), PR interval, and QRS duration. By-time-point and categorical analyses of ECG parameters were performed in the QT/QTcF analysis set, which included participants who had at least one dose of study drug, had baseline ECG measurements, and had at least one post-dose time point with a valid change from baseline in QTcF (ΔQTcF). The concentration-QTc analysis was performed in the PK/QTcF analysis set, which included (1) participants in the QT/QTcF analysis set who also had at least one evaluable plasma concentration and at least one matched post-dose plasma concentration-QTcF pair obtained at the same nominal time point, and (2) participants in the QT/QTcF analysis set who received placebo.

The primary cardiodynamic endpoint was ΔQTcF. The primary analysis evaluated the relationship between plasma concentrations of ceftibuten (*cis*- and/or *trans*-isomers) or ledaborbactam and/or ledaborbactam etzadroxil and ΔQTcF using concentration-QTc modelling, with the objective of excluding a placebo-corrected change from baseline in ΔQTcF (ΔΔQTcF) of ≥10 ms at clinically relevant plasma concentrations. Concentration-QTc analysis was performed using a linear mixed-effects model, with ΔQTcF as the dependent variable and plasma drug concentration (0 for placebo) as the primary predictor. Geometric mean maximum plasma concentration (C_max_) values were calculated separately within each dose group; concentrations were not pooled across dose levels. Centred baseline QTcF, defined for each participant as the individual baseline QTcF minus the population mean baseline QTcF, was included as a covariate, with time and treatment (active or placebo) as fixed effects, and participant-specific random intercepts and random concentration slopes.^10^ Bioanalytical methods, model details for the full model with two analytes, and data extraction details are summarized in the Supplementary Information (available as Supplementary data at *JAC* Online).

For ceftibuten, three models were evaluated: a full model including both *cis*-ceftibuten and *trans*-ceftibuten concentrations, a model including *cis*-ceftibuten alone, and a model including *trans*-ceftibuten alone. For ledaborbactam etzadroxil, three analogous models were evaluated: a full model including both ledaborbactam etzadroxil and ledaborbactam concentrations, a model including ledaborbactam etzadroxil alone, and a model including ledaborbactam alone. Model selection was based on the Akaike Information Criterion (AIC), which compares the relative fit of candidate models while penalizing unnecessary model complexity; the model with the lowest AIC was selected as the primary model for each drug.

Before model fitting, graphical exploratory analyses were performed to evaluate key assumptions, including absence of a drug effect on HR (Assumption 1), independence of QTcF from HR (Assumption 2), absence of hysteresis between plasma concentration and ΔΔQTcF (Assumption 3), and linearity of the concentration-QTc relationship (Assumption 4). Hysteresis was assessed using joint graphical displays of the least-squares (LS) mean ΔΔQTcF at each post-dose time point from the by-time-point analysis together with the corresponding mean plasma concentrations of ceftibuten (*cis*- and *trans*-isomers) or ledaborbactam and/or ledaborbactam etzadroxil. Following model fitting, the appropriateness of the selected pharmacodynamic model (linear versus nonlinear) was evaluated.

A by-time-point analysis of QTcF was performed as a secondary analysis using a mixed model for repeated measures with ΔQTcF as the dependent variable; treatment, time, and treatment-by-time interaction as fixed effects; and baseline QTcF as a covariate. Placebo-treated participants were analysed as a pooled group. LS mean ΔΔQTcF, standard error, and two-sided 90% confidence intervals (CIs) were estimated for each ceftibuten or ledaborbactam etzadroxil dose at each post-dose time point. The same model was used to analyse changes from baseline in HR, PR interval, and QRS duration.

### Sample size

For the ceftibuten study, the resulting sample size of 36 evaluable participants (27 receiving ceftibuten and nine receiving placebo) was retrospectively estimated to provide 72% power to exclude a QTc effect greater than 10 ms, as demonstrated by the upper bound of the two-sided 90% CI for the model-predicted ΔΔQTcF at the observed geometric mean C_max_. For the ledaborbactam etzadroxil study, the resulting sample size of 47 evaluable participants (35 receiving ledaborbactam etzadroxil and 12 receiving placebo) provided 82% power using the same approach. Statistical power was estimated using a two-sample *t*-test, assuming a one-sided significance level of 0.05, a true drug effect of 3 ms, and a standard deviation of ΔQTcF of 8 ms across the active treatment and placebo groups. Thus, the 72% and 82% values were not prospectively selected power targets but retrospective characterizations of the available datasets. Plasma concentrations were not inputs to these retrospective power calculations. The concentration-QTc models evaluated the full observed concentration range, with model-predicted effects reported at the observed dose-specific geometric mean C_max_ values. This calculation is conservative, because it does not account for increased precision obtained by analysing all paired PK and ECG observations from each participant using a linear mixed-effects model. Additional details of the power calculation have been reported previously.^11^ The concentration-QTc modelling approach is supported by the recommendations of Darpo *et al* and Ferber *et al*, and is consistent with experiences from many recent thorough QT (TQT) studies.^12,13^

## Results

### Participant characteristics

Demographic and baseline characteristics of the participants included in the concentration-QTc analyses are summarized in Table S1. These characteristics were generally similar between the active treatment and placebo groups within each study and were consistent with the healthy adult populations enrolled in the parent Phase I studies.^1,4^

### Baseline ECG parameters

For the ceftibuten study, baseline ECG data were available for all nine participants in each single-dose group and for nine pooled placebo participants across dose groups. For the ledaborbactam etzadroxil study, baseline ECG data were available for all six participants in each single-dose group, except for the 300-mg group, which included five participants; baseline data were also available for 12 pooled placebo participants across dose groups.

Baseline ECG parameters were consistent with those expected in a healthy adult population across all dose groups in each study. Among participants receiving ceftibuten or placebo, mean HR ranged from 69.6 to 73.2 beats per minute (bpm), mean QTcF from 401.7 to 406.6 ms, mean PR interval from 136.2 to 143.9 ms, and mean QRS duration from 102.4 to 106.0 ms. Among participants receiving ledaborbactam etzadroxil or placebo, mean HR ranged from 55.5 to 68.4 bpm, mean QTcF from 388.4 to 409.7 ms, mean PR interval from 130.5 to 156.0 ms, and mean QRS duration from 102.2 to 107.6 ms.

Ceftibuten administration was associated with a small, dose-dependent increase in HR during the first 3 hours after dosing, which was not considered clinically meaningful (Figure 1a). Mean ΔΔHR remained below 10 bpm for all dose groups and post-dose time points, with the exception of a value of 10.5 bpm at 3 hours in the 1200-mg group. This modest effect on HR was not considered sufficient to affect the validity of the concentration-QTc analysis. One participant each in the 800-mg, 1200-mg, and placebo groups met the predefined bradycardia outlier criteria (HR <50 bpm with >25% decrease from baseline) at a single time point. In contrast, ledaborbactam etzadroxil showed no dose-dependent effect on HR relative to placebo (Figure 1b). Mean ΔΔHR remained below 10 bpm for all dose groups and post-dose time points, indicating that ledaborbactam etzadroxil had no clinically relevant effect on HR.

**Figure 1.**
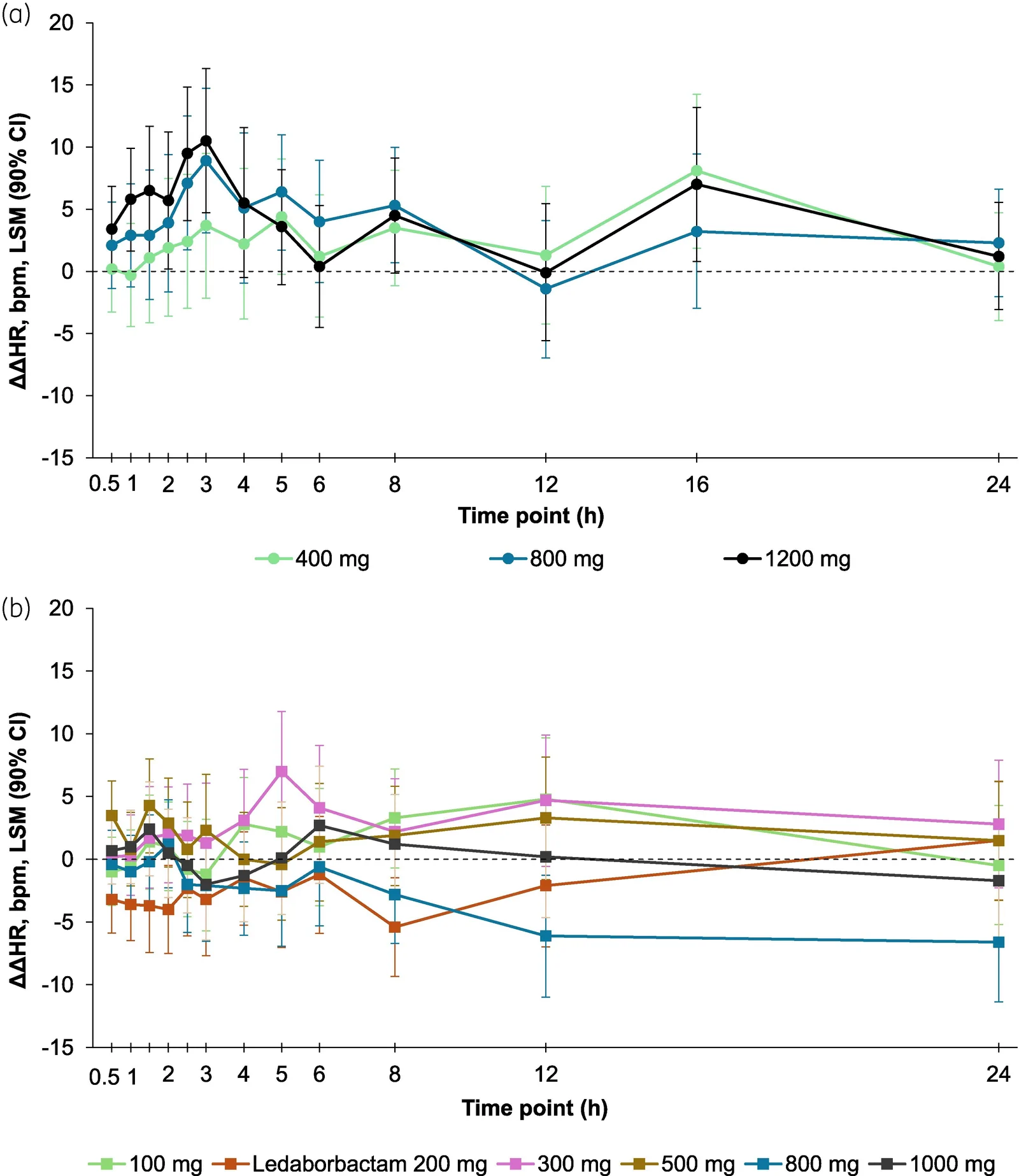
Placebo-corrected change from baseline HR (ΔΔHR, bpm) over time after administration of (a) ceftibuten and (b) ledaborbactam etzadroxil (QT/QTcF analysis set). bpm, beats per minute; HR, heart rate; LSM, least-squares mean; QTcF, heart rate–corrected QT interval using the Fridericia method.

### QTc analysis

Ceftibuten administration resulted in a pattern of LS mean change from baseline in QTcF over time that was similar to that observed with placebo (Figure 2a). The LS mean placebo-corrected changes from baseline in QTcF ranged from −5.4 ms (at 4 hours post-dose in the 1200-mg group and 16 hours post-dose in the 400-mg group) to 2.3 ms (at 24 hours post-dose in the 800-mg group), with no apparent dose-dependent relationship. No participant had a QTcF >450 ms or a change from baseline in QTcF >30 ms.

**Figure 2.**
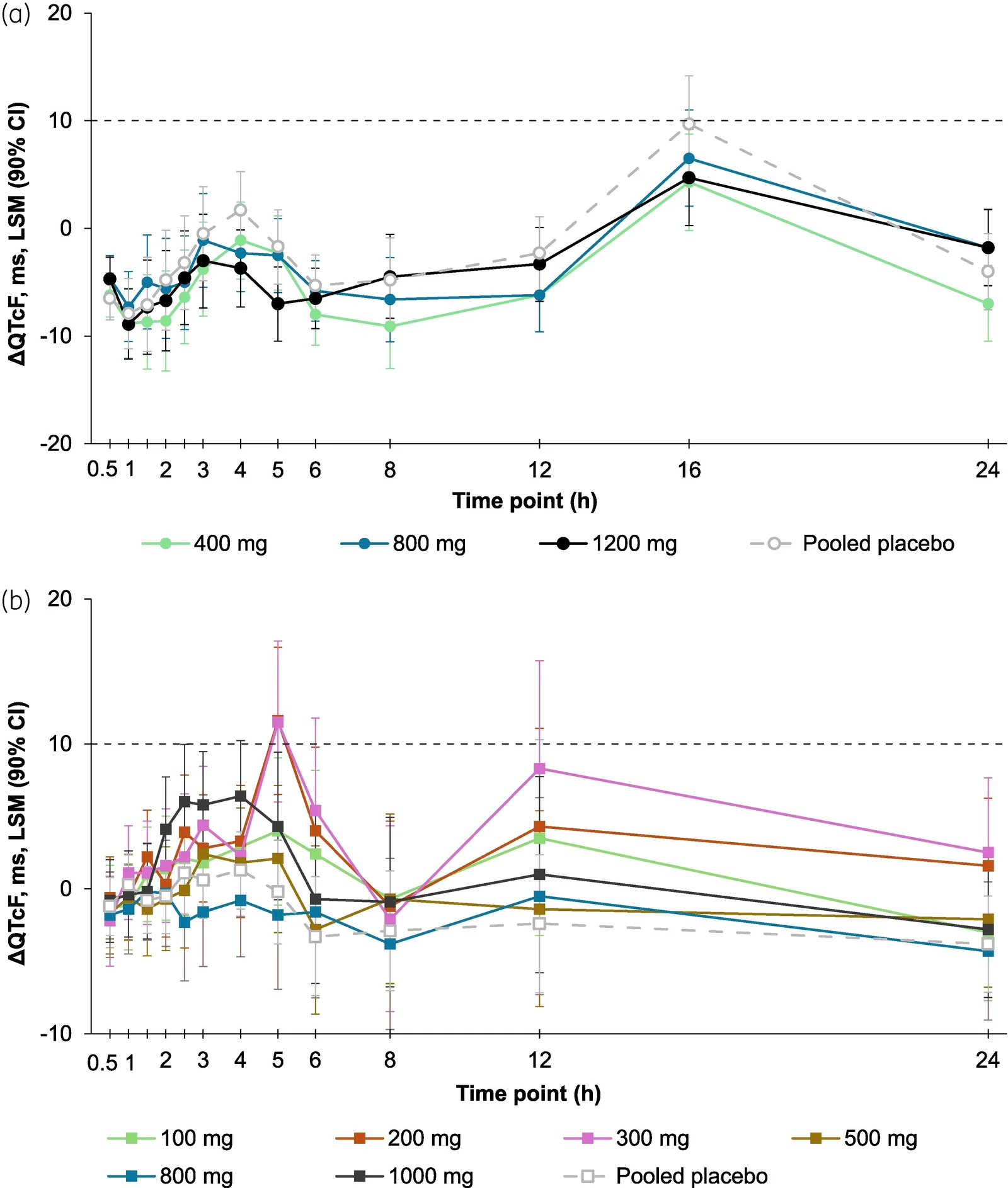
The effect of (a) ceftibuten or (b) ledaborbactam etzadroxil on QT interval over time (QT/QTcF analysis set): Change from baseline QTcF (ΔQTcF, ms). The black horizontal dashed line at 10 ms indicates a reference threshold for potential QT prolongation. CI, confidence interval; LSM, least-squares mean; QTcF, heart rate–corrected QT interval using the Fridericia method.

Ledaborbactam etzadroxil administration showed no dose-dependent effect on QTc, as assessed by the LS mean change from baseline in QTcF over time (Figure 2b). LS mean placebo-corrected changes from baseline in QTcF (ΔΔQTcF) ranged from −3.4 ms at 2.5 hours post-dose in the 800-mg group to 11.8 ms at 5 hours post-dose in both the 200-mg (90% CI, 5.58 to 18.02 ms) and the 300-mg (90% CI, 5.15 to 18.37 ms) group. These findings were not dose dependent, as the largest LS mean ΔΔQTcF was 2.3, 1.9, and 5.2 ms in the 500-, 800- and 1000-mg groups, respectively. No participant had a QTcF >480 ms in any dose group. One participant in the 300-mg group had a change from baseline in QTcF >30 ms and ≤60 ms at a single post-dose time point.

### Concentration-QT results

#### PK profile

Plasma concentration-time profiles of *cis*-ceftibuten, *trans*-ceftibuten, and ledaborbactam are shown in Figure 3. Mean *cis*-ceftibuten concentrations peaked between 2.5 and 4 hours post-dose across all dose groups (Figure 3a), whereas mean *trans*-ceftibuten concentration peaked at 4 hours post-dose at all dose levels (Figure 3b). For ledaborbactam, mean peak concentrations were observed at 1.5 hours post-dose in the 500-mg group and at 2 hours post-dose in the two highest-dose groups (800-mg and 1000-mg; Figure 3c). The dose-specific geometric mean C_max_ (range) of *cis*-ceftibuten was 16.9 (13.0–19.7), 27.5 (19.7–34.0), and 35.8 (26.6–43.4) µg/mL following ceftibuten doses of 400, 800, and 1200 mg, respectively. The geometric mean (range) C_max_ of *trans*-ceftibuten was 0.8 (0.5–0.9), 1.5 (0.8–1.9), and 1.8 (1.4–2.2) µg/mL following ceftibuten doses of 400, 800, and 1200 mg, respectively. For ledaborbactam, the dose-specific geometric mean (CV%) C_max_ was 4.0 (25.6), 6.0 (11.8), 7.6 (29.3), 16.8 (8.4), 24.0 (14.2), and 26.5 (7.9) µg/mL following doses of 100, 200, 300, 500, 800, and 1000 mg, respectively. For ledaborbactam etzadroxil, the dose-specific geometric mean (CV%) C_max_ was 0.17 (79.7), 0.17 (57.6), 0.35 (95.5), 0.92 (112), 1.16 (95.3), and 1.84 (21.2) µg/mL following doses of 100, 200, 300, 500, 800, and 1000 mg, respectively.

**Figure 3.**
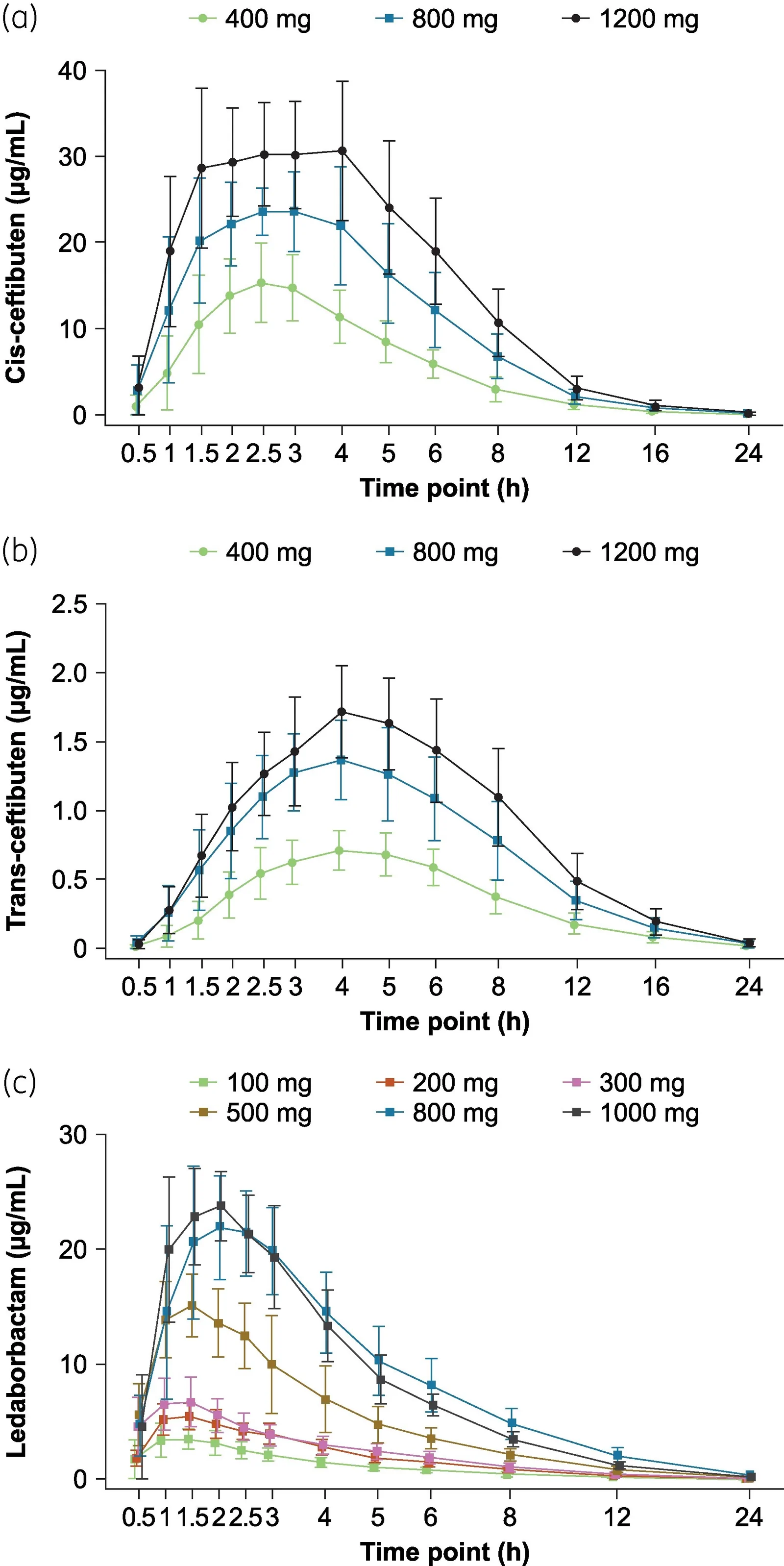
Plasma concentration-time profiles (PK/QTcF analysis set). (a) Mean *cis*-ceftibuten plasma concentrations over time; (b) mean *trans*-ceftibuten plasma concentrations over time; (c) mean ledaborbactam plasma concentrations over time. All PK samples were collected on day 1. PK, pharmacokinetics; QTcF, heart rate–corrected QT interval using the Fridericia method.

#### Linear concentration-QTc relationship

All four model-independent assumptions were satisfied. Assumptions 1 and 2 were met, as neither ceftibuten nor ledaborbactam had a clinically relevant effect on HR (Figure 1). Assumption 3 was also met for both ceftibuten and ledaborbactam etzadroxil, as no evidence of hysteresis was observed. Mean ΔΔQTcF across ceftibuten doses showed no relationship with plasma concentrations for either *cis*- or *trans*-ceftibuten. Similarly, hysteresis plots for ledaborbactam and ledaborbactam etzadroxil showed no apparent relationship between ΔΔQTcF and plasma ledaborbactam concentrations. Finally, Assumption 4 was met, as linear regression and locally weighted scatterplot smoothing (LOESS) curves showed good agreement across the observed concentration ranges for ledaborbactam and for *cis*- and *trans*-ceftibuten, except at very low concentrations (data not shown).

Although *cis*-ceftibuten is the more predominant and pharmacologically active isomer, the *trans*-ceftibuten model provided the best statistical fit according to AIC; therefore, it was selected as the primary model. Results for both isomers are presented because of the biological and clinical relevance of *cis*-ceftibuten. Predictions from alternative models were consistent with those from the primary models.

The concentration-QTc model for *cis*- and *trans*-ceftibuten alone are shown in Figure 4a and b, respectively. The concentration-QTc models for ledaborbactam and ledaborbactam etzadroxil alone are shown in Figure 5a and b, respectively. Model residuals were symmetrically distributed close to zero with approximately constant variance, indicating no apparent deviation from model assumptions (data not shown). The adequacy of the linear mixed-effects models for *cis*- and *trans*-ceftibuten are shown in Figure 4c and d, respectively, and for ledaborbactam and ledaborbactam etzadroxil are shown in Figure 5c and d, respectively. Goodness-of-fit plots demonstrated that observed mean placebo-adjusted ΔQTcF (90% CI) within each concentration decile was in close agreement with model-predicted ΔΔQTcF (90% CI) across the observed concentration ranges. Overall, the models adequately described the relationship between placebo-adjusted ΔQTcF and plasma drug concentrations, with decreasing ΔΔQTcF observed with increasing analyte concentrations. The concentration-QTc model results are summarized in Table S2. For all analytes, the upper bounds of the two-sided 90% CIs for the model-predicted ΔΔQTcF remained below 10 ms across the evaluated concentration ranges, excluding a clinically relevant QTc-prolonging effect.

**Figure 4.**
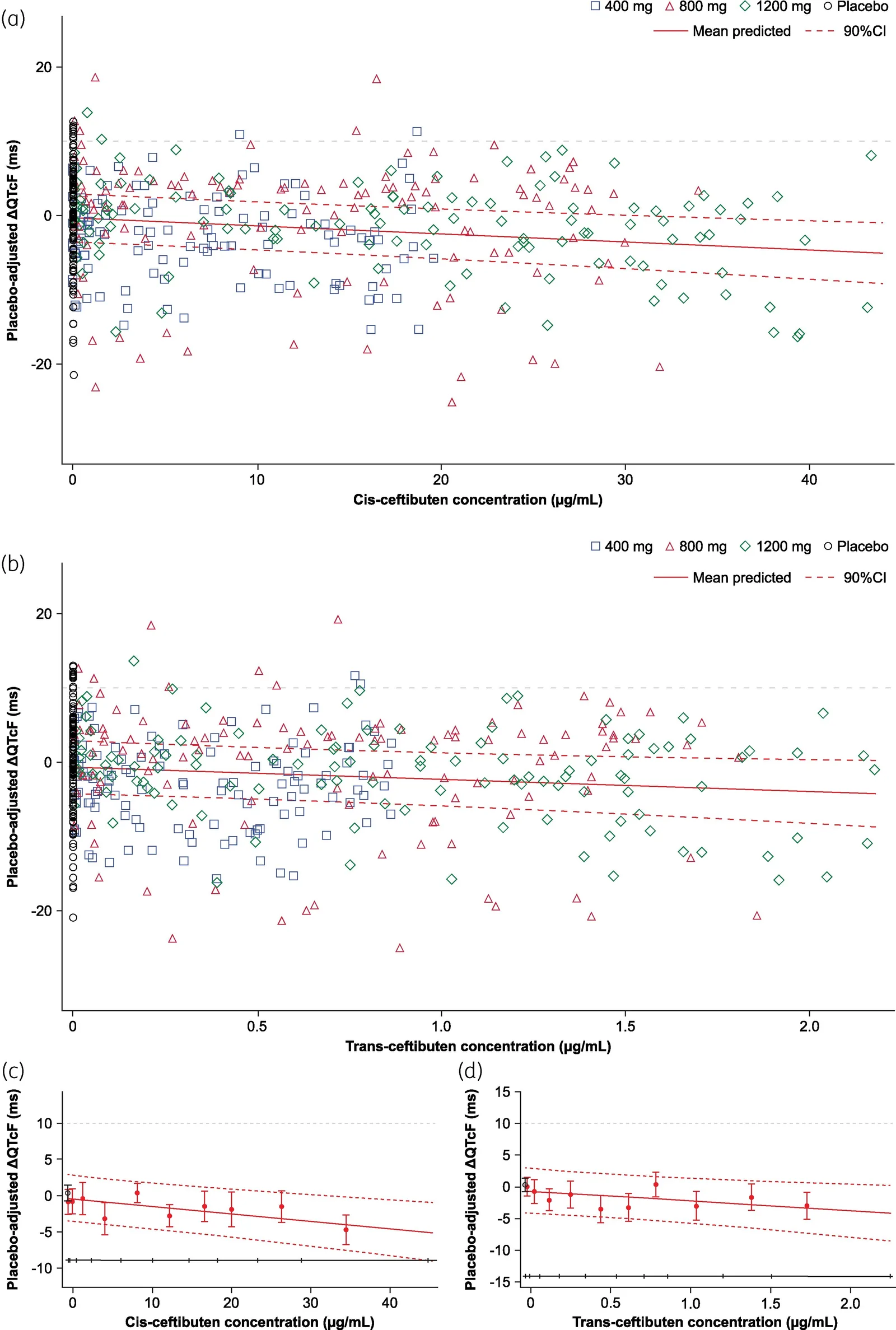
Cis- and *trans*-ceftibuten concentration-QTc analysis (PK/QTcF analysis set). Scatter plot of observed (a) *cis*-ceftibuten and (b) *trans*-ceftibuten plasma concentrations and estimated ΔΔQTcF, and model-predicted goodness-of-fit plots for (c) *cis*-ceftibuten and (d) *trans*-ceftibuten. In (c) and (d), the solid red line and the area in between the dashed red lines denote the model-predicted mean ΔΔQTcF with 90% CI. The red filled circles with the vertical bars denote the estimated mean ΔΔQTcF with 90% CI displayed at the associated median plasma concentration. The black circle with the vertical bars denotes the mean ΔΔQTcF with 90% CI for placebo at a concentration of 0. The horizontal black line with notches shows the range of concentrations divided into deciles where the area between each decile represents the point at which 10% of the data are present. The grey horizontal dashed line at 10 ms indicates a reference threshold for potential QT prolongation. CI, confidence interval; PK, pharmacokinetics; QTcF, heart rate–corrected QT interval using the Fridericia method.

**Figure 5.**
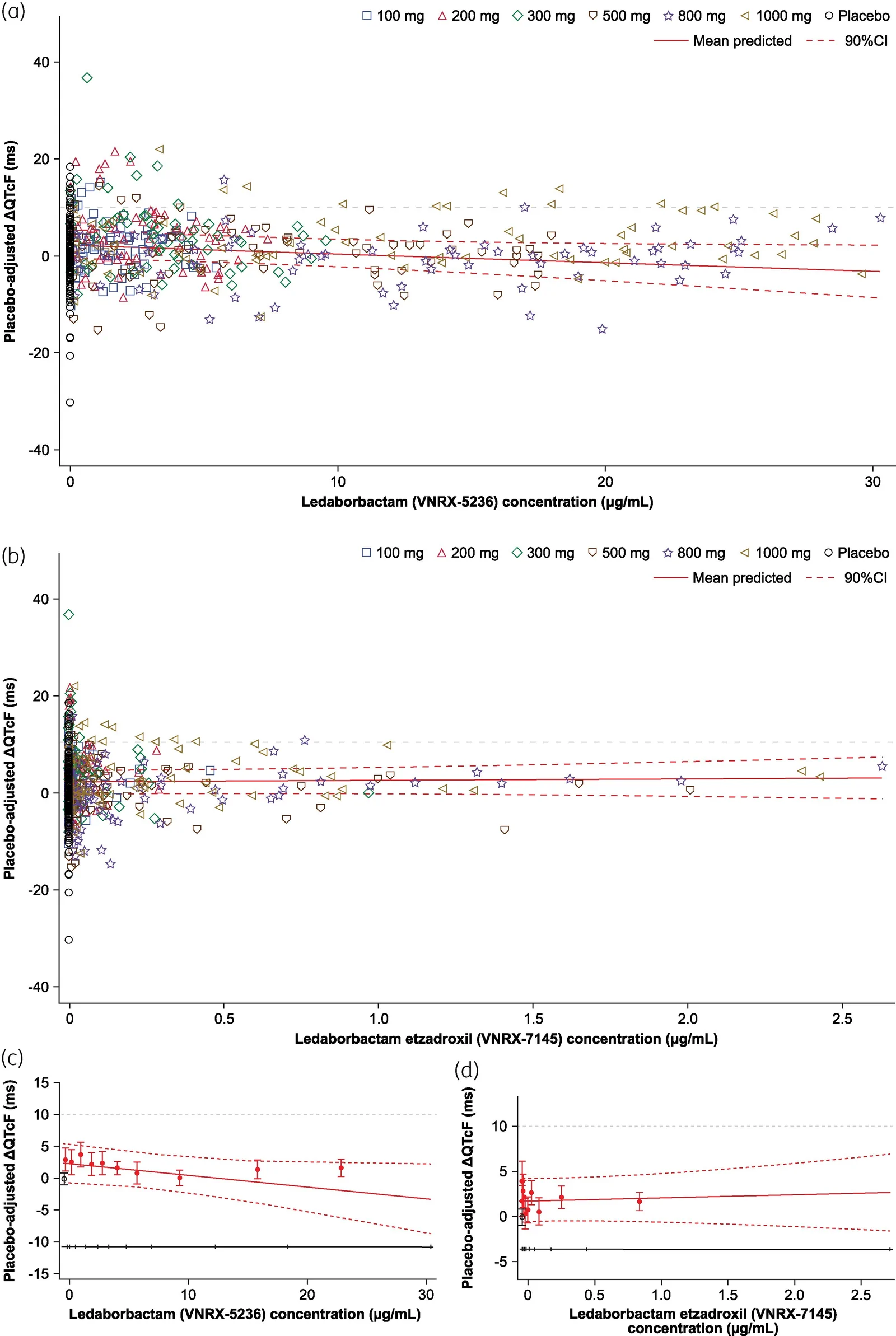
Ledaborbactam and ledaborbactam etzadroxil concentration-QTc analysis (PK/QTcF analysis set). Scatter plot of observed (a) ledaborbactam and (b) ledaborbactam etzadroxil plasma concentrations and estimated ΔΔQTcF, and model-predicted goodness-of-fit plots for (c) ledaborbactam and (d) ledaborbactam etzadroxil. In (c) and (d), the solid red line and the area in between the dashed red lines denote the model-predicted mean ΔΔQTcF with 90% CI. The red filled circles with the vertical bars denote the estimated mean ΔΔQTcF with 90% CI displayed at the associated median plasma concentration. The black circle with the vertical bars denotes the mean ΔΔQTcF with 90% CI for placebo at a concentration of 0. The horizontal black line with notches shows the range of concentrations divided into deciles where the area between each decile represents the point at which 10% of the data are present. The grey horizontal dashed line at 10 ms indicates a reference threshold for potential QT prolongation. CI, confidence interval; PK, pharmacokinetics; QTcF, heart rate–corrected QT interval using the Fridericia method.

Based on model predictions, a ΔΔQTcF effect exceeding 10 ms can be excluded up to plasma concentrations of 44 µg/mL for *cis*-ceftibuten and 2.2 µg/mL for *trans*-ceftibuten, and across the full observed concentration ranges for ledaborbactam and ledaborbactam etzadroxil (up to 30.3 µg/mL and 2.6 µg/mL, respectively).

### PR interval and QRS duration

Neither ceftibuten nor ledaborbactam had a clinically relevant effect on cardiac conduction, as assessed by changes in PR interval and QRS duration. For both treatments, LS mean changes from baseline in PR interval followed the pattern observed with placebo, and changes in QRS duration remained small throughout the observation period. For ledaborbactam etzadroxil, placebo-corrected changes from baseline in PR showed no evidence of dose dependence. Across all post-dose time points, placebo-corrected LS mean changes in QRS duration ranged from −2.7 to 0.6 ms for ceftibuten and remained within ±2.1 ms for ledaborbactam. No treatment-emergent T- or U-wave morphology abnormalities were observed following ceftibuten or ledaborbactam etzadroxil administration.

## Discussion

This study evaluated the potential for QT prolongation associated with ceftibuten and ledaborbactam etzadroxil using concentration-QTc modelling consistent with contemporary ICH E14 recommendations.^8^ Ceftibuten and ledaborbactam had no clinically relevant effect on cardiac conduction, supporting a low likelihood of clinically meaningful QTc liability at the anticipated clinical doses. Elevated ΔΔQTcF were observed after peak plasma concentrations at some lower ledaborbactam etzadroxil doses. Similar elevations were not observed at higher doses, and concentration-QTc analysis did not support a positive concentration-dependent relationship.

Based on concentration-QTc modelling, a placebo-corrected change from baseline in QTcF effect exceeding 10 ms can be excluded up to 44 µg/mL and 2.2 µg/mL for *cis*- and *trans*-ceftibuten, respectively, and up to 30.3 µg/mL and 2.6 µg/mL for ledaborbactam and ledaborbactam etzadroxil, respectively. These concentrations exceed the dose-specific geometric mean C_max_ values anticipated with the repeat-dose regimens evaluated in Phase I, which included ceftibuten 400 mg combined with ledaborbactam etzadroxil 300 or 500 mg every 8 hours.^4^ Because the fixed-dose combination remains investigational, the final therapeutic dose including the ratio of ceftibuten/ledaborbactam etzadroxil has not been established.

Ledaborbactam etzadroxil showed no clinically relevant effects on any ECG parameter, and ceftibuten had a small, transient, dose-dependent increase in HR that was not clinically relevant, further supporting an acceptable cardiac safety profile.

The PK and safety of ceftibuten and ledaborbactam etzadroxil, administered alone and in combination, were previously evaluated in two Phase I placebo-controlled studies (NCT04314206, VNRX-5024-101; NCT04243863, VNRX-7145-101).^1,4^ Both agents were well tolerated, and demonstrated linear, dose-proportional PK.^1,4^ Ceftibuten and ledaborbactam demonstrated a favourable PK profile at the higher doses that may be required for the treatment of infections caused by multidrug-resistant Enterobacterales.^1^ Coadministration of ceftibuten and ledaborbactam etzadroxil showed no clinically meaningful PK interaction and a safety profile consistent with that of ceftibuten alone.^4^

A limitation of these studies is that concentration-QTc evaluations were performed separately for ceftibuten and ledaborbactam etzadroxil, as the combination is under clinical development. As a result, a potential additive effect on QT prolongation cannot be fully excluded, although the combination did not increase ceftibuten or ledaborbactam C_max_ compared to dosing each individually. A further limitation is that the QT evaluation was conducted in healthy participants rather than the targeted patient population. The target population may be more susceptible to QTc prolongation than healthy individuals due to cardiovascular disease; electrolyte disturbances, particularly hypokalaemia; or concomitant medication. However, studies in the target population would present analytical challenges due to the number of variables that could affect the QT interval, and studies in healthy participants have greater internal validity, permit the detection and the exclusion of clinically relevant ECG effects, and are generally considered suitable for identifying drugs with a clinically relevant QT risk.^8^ Another limitation is lower power for ceftibuten (72%); however, β-lactams are not generally known for their QT liability, and the 82% power for a novel β-lactamase inhibitor such as ledaborbactam was sufficient to evaluate the QT interval prolongation for non-antiarrhythmic drugs.^11^

In summary, ceftibuten and ledaborbactam etzadroxil had no clinically relevant effect on ECG parameters, and neither demonstrated evidence of QT liability based on concentration-QTc analyses. Therefore, a clinically meaningful effect on QTc is not anticipated with the oral fixed-dose combination. These results support the cardiac safety profile of ceftibuten-ledaborbactam etzadroxil and indicate that a dedicated TQT study or additional QT evaluation is not necessary under current ICH E14 recommendations.

## Supplementary Material

dkag365_Supplementary_Data

## Supplementary data

Tables S1 and S2 are available as Supplementary data at *JAC* Online.
