# Supplementary material for: No clinically relevant QTc prolongation with ceftibuten or ledaborbactam etzadroxil: concentration-QTc analyses from two phase I studies
Source: J Antimicrob Chemother. 2026 Oct 23;81(11):dkag365. doi: 10.1093/jac/dkag365 (PMC13691604; doi:10.1093/jac/dkag365)
Supplement: Supplementary file 1 [file dkag365_supplementary_data.pdf]

Supplementary Information

Table S1. Demographic and baseline characteristics of the concentration-QTc analysis populations

Characteristic	Ceftibuten n = 27	Placebo n = 9	Ledaborbactam etzadroxil n = 35*	Placebo n = 12
Age, years, mean (SD)	28.8 (7.50)	24.3 (3.94)	33.5 (5.79)	33.8 (9.15)
Sex, n (%)				
Male	10 (37)	4 (44)	17 (49)	4 (33)
Female	17 (63)	5 (56)	18 (51)	8 (67)
Race, n (%)				
White	18 (67)	9 (100)	27 (77)	6 (50)
Black or African American	3 (11)	0	6 (17)	5 (42)
Asian	2 (7)	0	1 (3)	0
White + Black or African American	3 (11)	0	1 (3)	1 (8)
White + Asian	1 (4)	0	0	0
Ethnicity, n (%)				
Not Hispanic or Latino	26 (96)	9 (100)	11 (31)	7 (58)
Hispanic or Latino	1 (4)	0	24 (69)	5 (42)
BMI, kg/m^2, mean (SD)	23.59 (2.01)	23.32 (3.38)	26.43 (3.25)	27.45 (2.40)

Data are mean (SD) unless otherwise indicated. Height, weight, and BMI were determined at screening. The concentration-QTc analysis population included actively treated participants with at least one evaluable post-dose concentration-QTcF pair and participants who received placebo.

*One participant was excluded from the QT/QTc and PK/QTc analysis populations because no pre-dose ECG observations were available.

BMI, body mass index; ECG, electrocardiogram; PK, pharmacokinetic; QTcF, Fridericia-corrected QT interval; SD, standard deviation.

Table S2. Summary of concentration-QTc model parameters and predicted QTc effects

Parameter	Cis-ceftibuten	Trans-ceftibuten	Ledaborbactam	Ledaborbactam etzadroxil
Slope estimate (90% CI; units shown for each analyte)	−0.11 (−0.175 to −0.041) ms per mg/L	−0.0016 (−0.00331 to 0.00007) ms per µg/L	−0.00019 (−0.000382 to 0.000006) ms per ng/mL	0.00031 (−0.001203 to 0.001816) ms per ng/mL
Treatment-specific intercept, ms	−0.33	−0.59	2.46	1.79
Geometric mean C_{max}, µg/mL	**400 mg:** 16.9 **800 mg:** 27.5 **1200 mg:** 35.8	**400 mg:** 0.8 **800 mg:** 1.5 **1200 mg:** 1.8	4.01 to 26.50 across dose groups	0.17 to 1.84 across dose groups
Predicted ΔΔQTcF at geometric mean C_{max} (90% CI), ms	**400 mg:** −2.16 (−5.46 to 1.15) **800 mg:** −3.30 (−6.83 to 0.23) **1200 mg:** −4.19 (−7.97 to -0.41)	**400 mg:** −1.81 (−5.27 to 1.66) **800 mg:** −2.94 (−6.72 to 0.84) **1200 mg:** −3.43 (−7.44 to 0.59)	**800 mg**: −2.06 (−6.49 to 2.37) **1000 mg**: −2.53 (−7.35 to 2.30)	**800 mg**: 2.18 (−0.65, 5.00) **1000 mg**: 2.25 (−0.74, 5.23)
Highest evaluated concentration at which an effect >10 ms was excluded	~44 µg/mL	~2.2 µg/mL	30.3 µg/mL	2.6 µg/mL

AIC, Akaike Information Criterion; CI, confidence interval; C_{max}, maximum plasma concentration; ΔΔQTcF, change from baseline in Fridericia-corrected QT interval.

1. Bioanalytical methods

Total plasma concentrations of cis-ceftibuten, trans-ceftibuten, ledaborbactam, and ledaborbactam etzadroxil were quantified using validated liquid chromatography–tandem mass spectrometry (LC-MS/MS) methods. For cis- and trans-ceftibuten, 6-mL blood samples were collected into prelabelled, prechilled K_2EDTA tubes. The tubes were inverted 8 to 10 times, placed on ice, and centrifuged for 10 minutes at 1000 × g and 4 °C. Plasma was stored at −70 °C or below within 60 minutes of blood collection. Cis- and trans-ceftibuten were extracted from 0.100-mL plasma by protein precipitation followed by lipid filtration and quantified using a Sciex API 5000 LC-MS/MS system with an Ace 3 C18-PFP, 150 x 2.1 mm column and stable isotope-labelled $^{13}C_3,^{15}N_2$-ceftibuten as the internal standard. The monitored mass transitions were m/z 411.3→268.1 for cis- and trans-ceftibuten and m/z 416.0→273.0 for the corresponding internal standards. The calibration ranges were 150 to 75,000 ng/mL for cis-ceftibuten and 8.00 to 4000 ng/mL for trans-ceftibuten. Calibration curves were generated using weighted $1/x^2$ linear least-squares regression.[1]

For ledaborbactam and ledaborbactam etzadroxil, blood samples were collected into acidified (20 mM citric acid) sodium fluoride/potassium oxalate tubes. Following collection, the tubes were immediately placed on ice and centrifuged at 1300 x g for 10 minutes at 4 °C, and the resulting plasma was stored at −70 °C within 90 minutes after collection. Ledaborbactam and ledaborbactam etzadroxil were quantified in 0.100 mL of acidified plasma using validated LC-MS/MS methods. Samples were prepared using protein precipitation and analysed using a Sciex API 4000 equipped with a Kinetix 2.6 µM XB-C18, 2.1x50 mm column, with VNRX-7283 as the internal standard. The monitored mass transitions were m/z 262.1→142.0 for ledaborbactam, m/z 390.2→244.0 for ledaborbactam etzadroxil, and m/z 267.1→143.0 and 395.2→249.0 for the corresponding internal standards (VNRX-5643 and VNRX-7283, respectively). The calibration ranges were 10.0 to 10,000.0 ng/mL for ledaborbactam and 0.500 to 500 ng/mL for ledaborbactam etzadroxil. Separate calibration curves were generated for each analyte using weighted $1/x^2$ linear least-squares regression.[2]

For cis-ceftibuten, trans-ceftibuten, ledaborbactam, and ledaborbactam etzadroxil, inter-run accuracy ranged from −0.667% to 5.33%, intra-run accuracy ranged from −5.33% to 6.33%, inter-run precision was ≤6.61%, and intra-run precision was ≤4.19%. The assays also met prespecified acceptance criteria for selectivity, matrix effects, recovery, batch size, carryover, and dilution integrity.

Cis- and trans-ceftibuten were stable in plasma for 328 and 305 days at −80 °C and after five freeze-thaw cycles, respectively. Ledaborbactam and ledaborbactam etzadroxil were stable in acidified plasma for 931 and 250 days at −80 °C and after five freeze-thaw cycles, respectively. All study samples were stored and analysed within the conditions and durations for which analyte stability had been demonstrated.

2. Model details

Model details for the full model with two analytes are as follows: $\Delta QTcF_{ijk} = \alpha \times TRT_j + \beta_k \times t_k + \eta_{\mu,i} + (\theta_1 + \eta_{c1,i})\ CONC1_{ijk} + (\theta_2 + \eta_{c2,i})\ CONC2_{ijk} + \gamma \times B_{QTc,i} + \varepsilon_{ijk}$, where i is the i-th participant; j is the j-th treatment; k is the k-th time point; TRT_j is the j-th treatment effect (active = 1 or placebo = 0); t_k is the k-th time effect; $CONC1_{ijk}$ and $CONC2_{ijk}$ are the concentrations of analyte 1 and analyte 2 at the k-th time point for treatment j for participant i, respectively; $B_{QTc,i}$ is the centred baseline QTcF for the i-th participant; α and β_k are the fixed effects for treatment and time, respectively; θ_1, θ_2, and γ are the fixed effects for the slopes of concentrations and centred baseline QTcF; $\eta_{\mu,i}$, $\eta_{c1,i}$, and $\eta_{c2,i}$ are the participant-specific random effects for the intercept and slopes, respectively, having mean [0,0,0] and unstructured covariance matrix; and ε are independent residual errors having mean zero and variance σ^2. The model-predicted placebo-corrected ΔQTcF (ΔΔQTcF) at specified concentration levels was calculated as: $\Delta\Delta QTcF = \alpha + \theta_1 \times CONC1 + \theta_2 \times CONC2$.[3,4] For the individual models with a single analyte, only one term of $(\theta_1 + \eta_{c1,i})\ CONC1_{ijk}$ and $(\theta_2 + \eta_{c2,i})\ CONC2_{ijk}$ was retained in the model.

3. Data extraction

In the ceftibuten study, plasma PK samples were collected before dosing and at 0.5, 1, 1.5, 2, 2.5, 3, 4, 5, 6, 8, 12, 16, and 24 hours post-dose.[1] Twelve-lead ECGs were extracted at three prespecified pre-dose time points (−45, −30, and −15 minutes) and at each of the 13 post-dose PK sampling time points. Thus, each actively treated participant contributed 13 time-matched post-dose concentration–Fridericia-corrected QT interval (QTcF) pairs. In the ledaborbactam etzadroxil study, plasma PK samples were collected before dosing and at 0.25, 0.5, 0.75, 1, 1.5, 2, 2.5, 3, 4, 5, 6, 8, 12, 16, 24, 36, and 48 hours post-dose; the 0.25- and 0.75-hour samples were collected only in the 1000-mg cohort.[2] Twelve-lead ECGs were extracted at the three prespecified pre-dose time points and at 0.5, 1, 1.5, 2, 2.5, 3, 4, 5, 6, 8, 12, and 24 hours post-dose. Thus, each evaluable actively treated participant contributed 12 time-matched post-dose concentration-QTcF pairs. For both studies, PK samples collected at time points without corresponding ECG extractions were used for PK characterisation but were not included in the concentration-QTc analysis. The concentration-QTc analyses therefore used serial concentrations spanning the absorption, peak-exposure, and elimination phases rather than only trough or peak concentrations. At each nominal ECG time point, up to 10 non-overlapping ECG replicates were extracted from the continuous Holter recordings using thorough QT (TQT) Plus methodology and analysed using the early precision QT technique.[5] All interpretable cardiac cycles were assessed according to predefined quality metrics, and low-confidence beats were manually reviewed and adjudicated.

References

1. Dorr MB, de Oliveira CF, van de Wetering J *et al*. Pharmacokinetics and safety of single and repeat doses of ceftibuten in healthy participants: a phase 1 dose escalation study. *Antimicrob Agents Chemother* 2025; **69**: e0008725. https://doi.org/10.1128/aac.00087-25
2. de Oliveira CF, Dorr Mary B, van de Wetering J *et al*. Safety and pharmacokinetics of single and multiple doses of ledaborbactam etzadroxil with or without ceftibuten in healthy volunteers. *Antimicrob Agents Chemother* 2025; **69**: e00210–25. https://doi.org/10.1128/aac.00210-25
3. Garnett C, Bonate PL, Dang Q *et al*. Scientific white paper on concentration-QTc modeling. *J Pharmacokinet Pharmacodyn* 2018; **45**: 383–97. https://doi.org/10.1007/s10928-017-9558-5
4. Garnett C, Bonate PL, Dang Q *et al*. Correction to: Scientific white paper on concentration-QTc modeling. *J Pharmacokinet Pharmacodyn* 2018; **45**: 399. https://doi.org/10.1007/s10928-017-9565-6
5. Darpo B, Fossa AA, Couderc JP *et al*. Improving the precision of QT measurements. *Cardiol J* 2011; **18**: 401–10.
